# Induction of high numbers of T_reg_ cells post treatment with anti-IL-2/IL-2 complex associates with alleviation of experimental psoriasis-like skin inflammation

**DOI:** 10.1186/s12865-025-00716-5

**Published:** 2025-11-18

**Authors:** Samar Salman, Sohaila M. Khalil, Amany Mohammed Abdel-Latif, Yasmina Ahmed El Attar, Mohamed Labib Salem

**Affiliations:** 1 Department of Dermatology and Venereology, Faculty of Medicine, Tanta University Hospital, Tanta, Egypt; 2 Immunology and Biotechnology Unit, Department of Zoology, Faculty of Science, Tanta University, Tanta, Egypt; 3 Center of Excellence in Cancer Research, Tanta University Teaching Hospital, Tanta, Egypt

**Keywords:** CD4, CD25, Foxp3 IL-2, Imiquimod, Inflammation, Psoriasis, T_reg_ cells

## Abstract

**Background:**

Psoriasis is a prevalent autoimmune skin disorder; however, the mechanism of its pathogenesis remains fully understood. The imbalance of regulatory T (T_reg_) cells and effector T cells represents one potential mechanism, where a low dose of IL-2 is important.

**Aim of the work:**

Given that IL-2/IL-12 complex is considered as an immune modulator for antigen-activated lymphocyte proliferation, this study aimed to compare the immunophenotypic, clinical, and histological effects of anti-IL-2/IL-2 complex to a low dose of free IL-2 on experimental psoriasis-like skin inflammation induced by imiquimod.

**Materials and methods:**

Thirty-five Balb/c male mice were left without treatment, or were received topical application of imiquimod (IMQ, 3.125 mg/mouse) to induce psoriasis-like skin inflammation, and then the mice were treated with intraperitoneal (i.p.) injection of 100 µL containing anti-IL-2/IL-2 complex (2.5 µg /0.5 µg/mouse), or topical steroids (62.50 mg/mouse), or low dose of free IL-2 (i.p.; 0.5 µg/mouse). The expression levels of CD4, CD25, and Foxp3 in the leukocytes were assessed by multiparametric flow cytometry. The effects of different treatments on the histology and pathology of the induced psoriasis were also assessed.

**Results:**

IMQ-induced hyperkeratosis, parakeratosis and mild papillomatosis with the retained nuclei in the keratin layer, whereas acanthosis with exocytosis was prominent in the epidermal layer. Lymphocyte infiltration was profusely all over the dermis. Additionally, there were some degrees of Munro micro abscesses were observed in the keratin layer with a collection of neutrophils in the group treated with standard betamethasone cream which showed mild improvement clinically, histopathological with no significant difference between this group and the naïve and positive control groups. After 7 days from the onset of treatment, we found that treatment of mice with anti-IL-2/IL-2 complex decreased the thickness of the epiderms as compared to their groups. Furthermore, the relative number of CD4^+^Foxp3^+^CD25^+^ cells showed increases in psoriasis mice treated with anti-IL-2/IL-2 complex as compared to other groups.

**In conclusion:**

Anti IL-2/IL-2 complex therapy effectively ameliorated the clinical manifestations of psoriasis, with no apparent side effects, providing a new strategy for treating psoriasis.

## Introduction

Psoriasis vulgaris is a common, chronic, relapsing autoimmune-mediated skin disease, clinically characterized by well-defined, scaly red plaques [1]. It is a common chronic inflammatory skin condition Its is considered as a systemic inflammatory state which is seen in approximately 85% of cases and commonly manifests as erythematous plaques with thick scaling on the extensor surfaces, trunk, and scalp [2, 3] that is associated with systemic production of pro-inflammatory/inflammatory cytokines and chemokines, causing organ dysfunction and subsequently results in in various comorbidities. As such, it is necessary to identify and treat these comorbidities to extend life expectancy and enhance quality of life [4]. The intimate connection between the epithelial immunological microenvironment (EIME) and immune-mediated inflammatory skin disorders has been the subject of intense attention in recent years [5]. Immune cells should cooperate under normal circumstances, but in psoriasis, their differentiation and proliferation are dysregulated, which causes keratinocytes to mature rapidly and arrange on the skin’s surface to create characteristic scaly plaques and secreting proinflammatory cytokines [6].

Although the pathogenesis of psoriasis is not yet fully explained, understanding the interaction between the innate and adaptive immune systems has enabled researchers to find new biomarkers that can be used to predict treatment response and open the door to personalized treatments. The identification of particular cytokine circuits including IL-6, IL-17 A, IL17 F, IL-22, IL-23, and TNF-α,) indicate that these mediators interact with each other, forming a network that is likely initiate psoriasis [7, 8]. This could explain why imiquimod, a TLR7/8 agonist and a potent inducer of these cytokines, could induce psoriasis mouse model by its topical application on mouse skin through induction of IL-23/IL-17 axis [9]. IMQ is a synthetic drug with immunomodulating action that is a toll like receptor 7 (TLR7) ligand that approved by FDA. Imiquimod is a topical treatment on the face and scalp; it is available as 2.5%; 3.75% (Zyclara^®^) and 5% cream (Aldara^®^). It activates innate immune cells (e.g. macrophages, dendritic cells, and natural killer cells), inducing them to release of cytokines IFN-α, IFN-γ, TNF-α, IL-6, and IL-12. These cytokines mediate the activation of cytolytic T-cells that are responsible for the lysis of target tumor cells [8]. The IMQ-induced mouse model is one of the most often used animal models that mimic psoriasis. This illustration may be suitable for testing topical therapy alternatives since it replicates several features of the intricate cutaneous pathophysiology. Psoriasis is mainly caused by immune dysregulation, in particular hyperfunction of T helper 17 (Th17) cells [10]. Normally, immune homeostasis is maintained by T_reg_ that inhibits immune effectors including Th17 cells [11]. In this regard, it has been reported that psoriasis is associated with impaired suppressive function of T_reg_ cells [12]. Therefore, one of the potential mechanisms for psoriasis treatment could be by increasing the numbers of T_reg_ cells as suggested previously [13]. In line with this notion, in vitro selective expansion of T_reg_ cells has shown potential effects in evoking immunosuppression against several autoimmune diseases as well as in the setting of organ transplantation [14]. Although effective, however, the expansion of T_reg_ cells is limited by the numbers during their generation in vitro and their survival upon their injection in vivo. Further, the quality of these cells varies depending on the expansion protocol. Therefore, approaches for in vivo expansion of T_Reg_ cells can be considered as an alternative approach to ex vivo expansion strategy as proposed previously [15]. Given that T_reg_ cells express IL-2 receptors [16], one potential approach for in vivo expansion of T_reg_ cells is based on the administration of IL-2 [17].

IL-2, as a pleiotropic cytokine, can have both autocrine and paracrine immunomodulatory effects [18]. However, its beneficial effects on the cell survival and growth, and their lineage stability depend on the type of cell and the context of the immune microenvironment [19]. Upon concomitant stimulation of the T cell receptor (TCR) andCD28, signal 1 and 2, respectively, in conventional CD4 ^+^ T cells either in vitro or in vivo, these cells produce a significant amount of IL-2 [20]. Although in smaller amounts, IL-2 can also be produced by other immune cells like dendritic cells, NK cells, and CD8 T cells [11]. Since FOXP3 represses IL-2 transcription in conjunction with other transcription factors, T_reg_ are heavily reliant on external IL-2 supplies [17]. This would explain the efficacious capability of exogenous administration of IL-2 to expand T_reg_ cells in vivo [11, 21]. IL-2 promotes the expression of FOXP3, the master transcription factor required for T_reg_ stability and suppressive function [22]. Low-dose IL-2 therapy is being explored to selectively boost T_reg_ while avoiding excessive activation of conventional T cells, which can cause inflammation [23]. IL-2 mutants or IL-2 complexes designed to preferentially stimulate T_reg_ are being tested in autoimmune diseases, graft-versus-host disease (GVHD), and transplantation tolerance [11, 24].

Although IL-2 has been though previously as an inflammatory cytokine and its presence is responsible for the autoimmune reaction [11], a new role of this cytokine emerged after discovering that IL2 gene knockout in mice caused the flourishing of the reaction [25], indicating to its double-edged sword on the quality of the resultant immunity through expansion of T_reg_ cells [26]. Furthermore. accumulated evidence has shown that low dose of IL-2 alone or complexed with anti-IL-2 monoclonal antibodies (mAbs) can have a potential effect to inducing activation and expansion of T_reg_ cells [27]. Therefore, our hypothesis in the present study is that anti-IL-2/IL-2 complex in psoriasis-like model will expand higher T_reg_ cells in vivo than free IL-2, resulting in mitigation of the associated inflammation. Therefore, we aimed to compare the immunomodulatory effects of anti-IL-2/IL-2 complex to free IL-2 on induced psoriasis-like skin inflammation induced in mice by the TLR7 agonist imiquimod.

## Materials and methods

### Animals

Male Balb/c mice (20–23 g) were obtained from Misr University for Science and Technology and housed in acrylic cages at The Animal Facility, Zoology Department, Faculty of Science, Tanta University according to the rules of The National Council of the National Academics x. The experiments were approved University (Protocol #32082/01/18) by the Institutional Ethical Committee for the Use of animals in research, Faculty of Science, Tanta University. The mice were acclimatized for 3 days before experimentation. The mice were euthanized using CO_2_ followed by cervical dislocation. Using a non-pre-charged chamber, CO_2_ is dispensed from a commercial cylinder with fixed pressure regulator and inline restrictor controlling gas flow within 30–70% of the chamber volume per minute to comply with American Veterinary Medical Association (AVMA) guidelines for the euthanasia of animals.

### Reagents

Imiquimod (Aldara^®^) was purchased from (Aldara, 3M Pharmaceuticals, Cairo, Egypt) with each sachet containing 250 mg of imiquimod (IMQ). Recombinant mouse IL-2 (rmIL-2, Cat #: 575406) and purified anti-IL-2 (Cat #: 503704) were purchased from BioLegend (USA, SanDiego, CA92121). Anti-IL-2/IL-2 complex was prepared by mixing 2.5 µg anti-IL-2 with 0.5 µg IL-2 as previously described [28]. Anti-IL-2/IL-2 complexes were incubated at 37 °C for 30 min before their injection. Anti-mouse mAbs, including anti-CD4 PerCP Cy5.5, anti-CD25 FITC, and anti-FOXP3 APC, for flow cytometry were purchased from (BD Biosciences, San Jose, CA, USA, FACS lysing solution was purchased from (BD Biosciences, San Jose, CA, USA), and phosphate-buffered saline (PBS) was purchased from (FIPCO, El Fath for Pharmaceutical & Cosmetics Industries, Alexandria, Egypt). FcR blocking reagent was purchased from (Miltenyi Biotec, Bergisch Gladbach, Germany).

### Induction of psoriasis-like lesion

IMQ was used to induce psoriasis-like skin lesions in mice as previously described [29]. Briefly, the dorsal skin of the mice was shaved and a commercially available 5% IMQ cream was topically applied on the shaved area daily topical at a dose of 62.50 mg for six successive days. This topical treatment is equal to a daily dose of 3.125 mg of the active ingredient (IMQ). The psoriasis-like lesion was confirmed by the presence of typical findings in H&E-stained sections, including epidermal hyperplasia, parakeratosis, Munro microabscesses, and an inflammatory infiltrate.

The inflammation was scored as mentioned below [30].

### Scoring severity of skin inflammation

Scoring system based on the clinical Psoriasis Area and Severity Index (PASI) was created to rate the degree of back skin inflammation; however, for the mouse model, the affected skin area is not included in the final result. On a scale of 0 to 4, erythema, scaling, and thickening were each given a score: 0 for none, 1 for minor, 2 for moderate, 3 for marked, and 4 for extremely marked. A grading chart with red tints was used to determine the degree of erythema. On a scale of 0 to 12, the cumulative score—which consists of erythema, scaling, and thickening—was used to gauge the degree of inflammation. Skin thickness was measured using digital caliper. An increase in skin thickness indicated epidermal proliferation and inflammation [30].

### Treatment of mice with anti-IL-2/IL-2 complex

On day 7 of IMQ treatment was applied stimultaneously. only mice that showed psoriasis-like lesion were selected for treatment with IL-2 complexes or with betamethasone as a conventional drug. For treatment with free IL-2, mice were treated with intraperitoneal injection (i.p.) of 0.5 µg IL-2 per day for three consecutive days. For treatment with IL-2 complexes, mice were treated with i.p. injection of anti-IL-2/IL-2 complex (2.5 µg anti-IL-2 + 0.5 µg IL-2) daily for three successive days. For treatment with the conventional anti-inflammatory drug, mice were treated topically with betamethasone valerate (BMV), a medium-potency corticosteroid, applied once a daily for five days for group III.

**Fig. 1:**
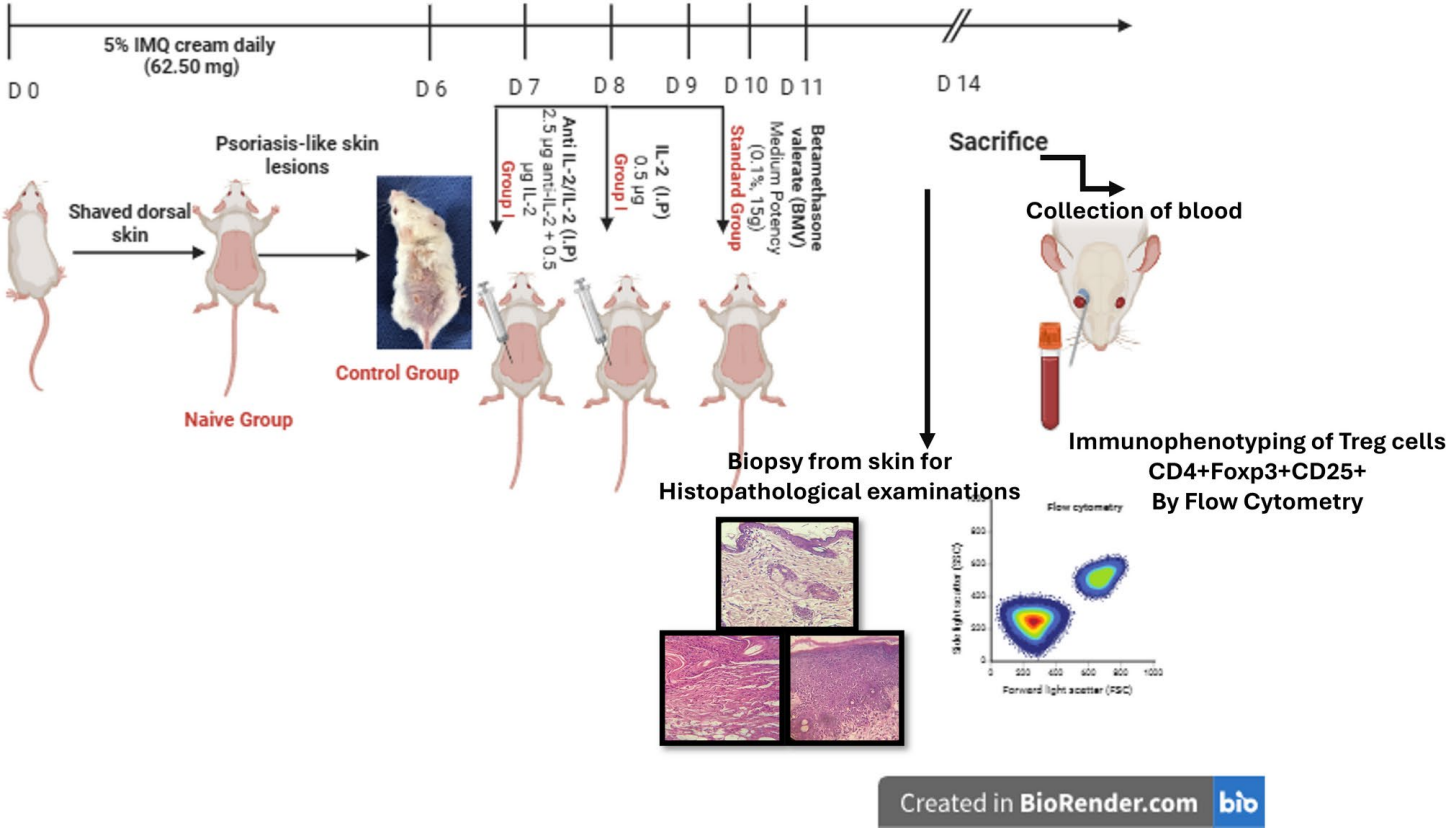
The induction and treatment of psoriasis-like skin lesions: The process begins with the topical application of Aldara (Imiquimod) cream, leading to the development of psoriasis-like lesions over seven days. Two treatment protocols are outlined: (1) administration of an anti-IL-2/IL-2 complex for three days and (2) administration of a low dose of IL-2. After seven days of treatment, clinical, histopathological, and immunological assessments are conducted, including T regulatory cell measurements via flow cytometry. The timeline also indicates the point of sacrifice and blood collection for further analysis

Finally, after another four days with sole use of imiquimod cream, mice in all groups were sacrificed and the blood samples and skin biopsies were collected, as shown in Fig. 1.

### Collection of peripheral blood samples

Peripheral blood samples (1 mL) were collected from the retroorbital sinus of the mice in tubes with ethylenediamine tetraacetic acid (EDTA). The tubes were centrifuged at 500 rpm for 5 min at 4°C. These blood samples were used for multiparametric flow cytometry analysis.

### Histopathological examinations

Tissue samples were collected at baseline and after seven days, then fixed in 10% neutral-buffered formalin for optimal preservation. Following fixation, the samples were processed and embedded in paraffin blocks. Thin sections, 4 μm in thickness, were prepared using a rotary microtome and subsequently stained with hematoxylin and eosin (H&E) as previously described [30]. The stained sections were then examined under a microscope equipped with a digital camera system after 7 days of Aldara treatment and on the day of sacrifice following 7 days of treatment.to assess histopathological changes.

### Morphometric analysis of the mean thickness of the epidermis

**Fig. 2:**
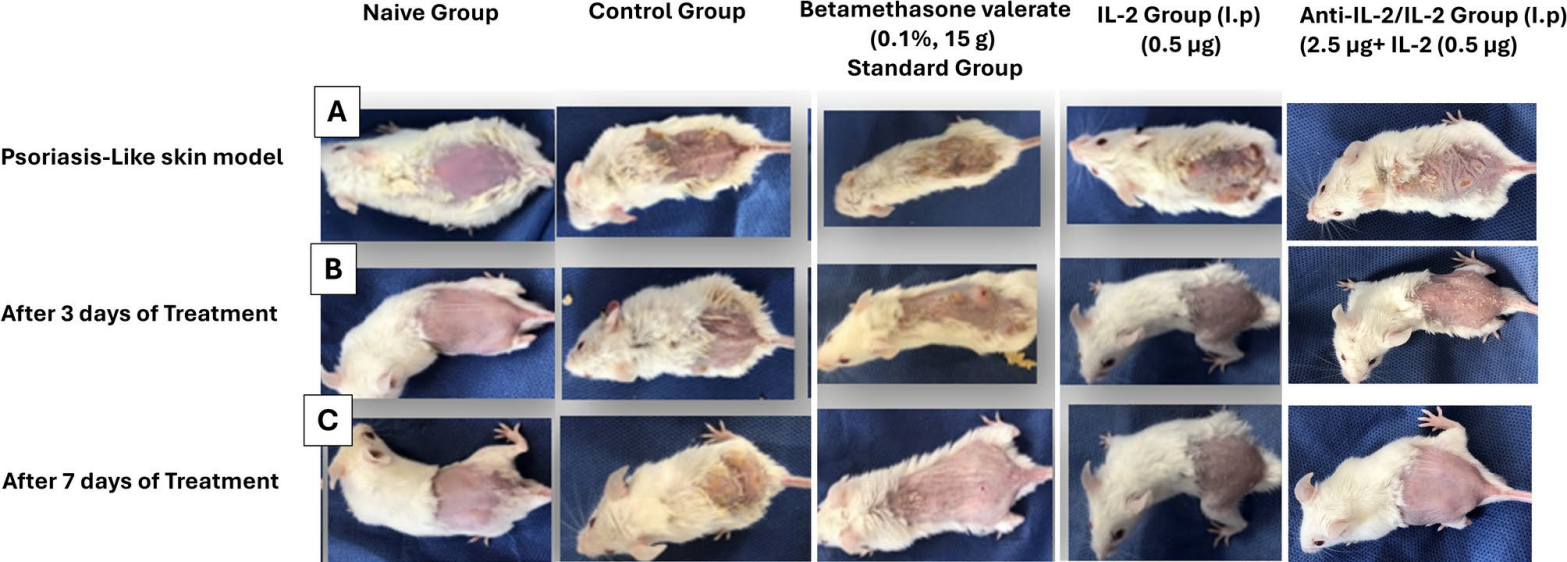
A comparative analysis of psoriasis-like skin lesions in different mouse model groups across various treatment stages. Panel **A** illustrates the psoriasis-like skin condition before treatment, while Panel **B** shows the progression after three days of intervention. Panel **C** displays the effects after seven days of treatment. The study evaluates different therapeutic approaches, including an anti-IL-2/IL-2 complex, IL-2 alone, topical corticosteroids, a control-positive group, and a naïve group. Visual differences in lesion severity and healing suggest the varying efficacy of treatments in modulating disease progression

For histomorphometry measurements, each H&E prepared skin biopsy specimen was randomly selected and photographed at different fields with a 10× objective. In each photomicrograph, the thickness of the epidermis was measured with ImageJ software. Three measurements for each mice sample were taken and their average was used in the statistical analysis as shown in Fig. 2.

### Immunophenotyping of T_reg_ cells by flow cytometry

The peripheral blood samples were surface-labeled with anti-mouse CD4 PerCP Cy5.5, CD25 FITC by using concentrations recommended by the manufacturers of each antibody. The cells were further fixed and permeabilized using a permeabilization buffer and then stained with anti-mouse Foxp3 APC. Briefly, the stained samples were incubated in the dark for 20–30 min. Samples were then centrifuged at 1500 rpm for 5 min, then the supernatant was discarded to remove the lysed RBCs. Cells were washed twice using PBS to remove any remaining debris or RBCs, and then cells were suspended in PBS. Negatively stained samples were used as internal controls all over the experiments. Data were acquired on a FACS Canto cytometer (BD Bioscience, Franklin Lakes, NJ) and analyzed on DIVA software.

### Statistical analysis

Data was fed to the computer and analyzed using IBM SPSS software package version 20.0. (Armonk, NY: IBM Corp) The Kolmogorov-Smirnov test was used to verify the normality of distribution. Quantitative data were described using range (minimum and maximum), mean, standard deviation and media. The significance of the results obtained was judged at the 5% level.

The tests used were F-test (ANOVA) and Bonferroni correction, for normally distributed quantitative variables, to compare between more than two groups, and Post Hoc test (Tukey) for pairwise comparisons.

## Results

### Phenotype change of the experimental mice “clinically”

**Fig. 3:**
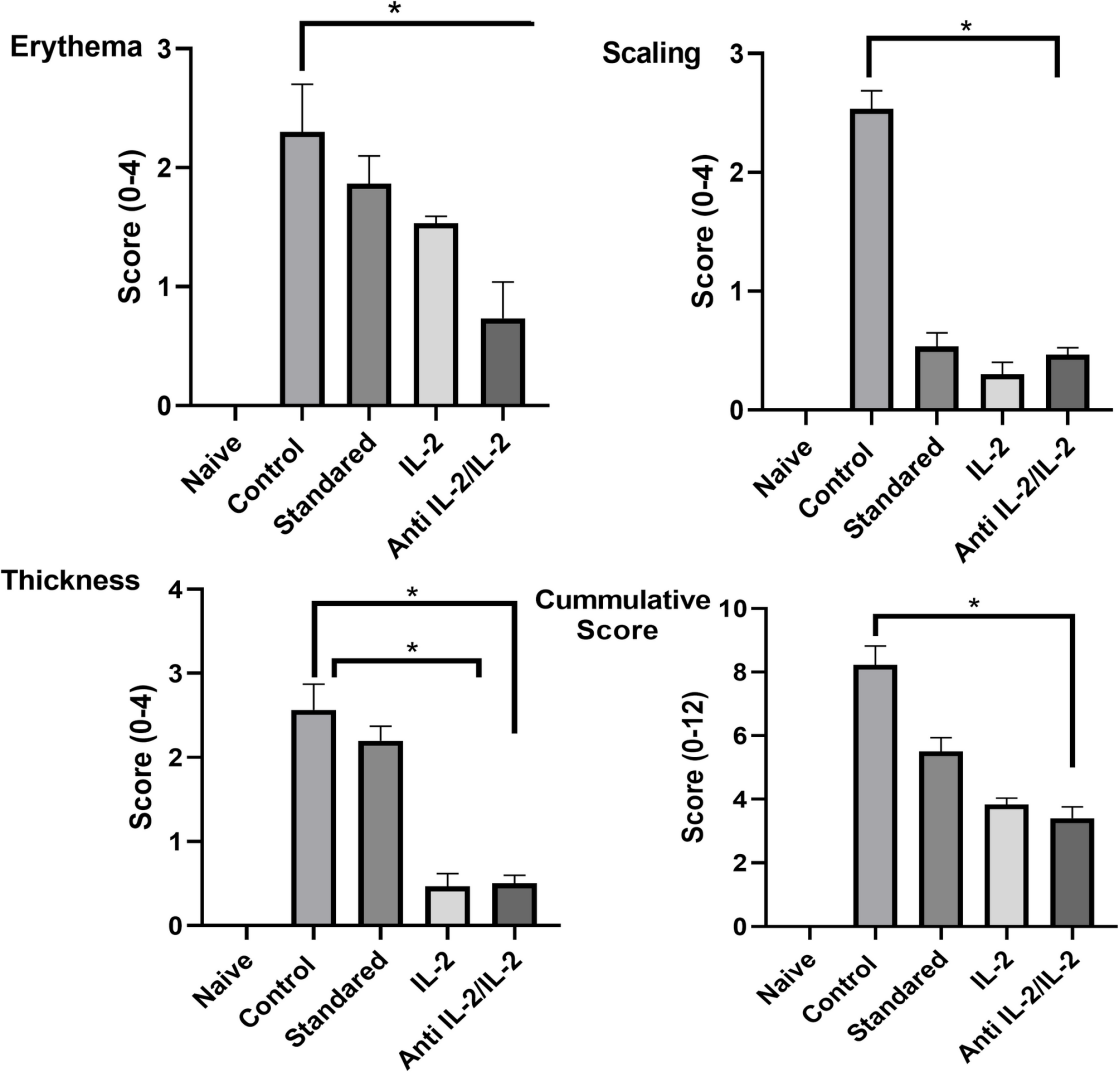
Phenotype change resembles psoriasis of the experimental mice “clinically” after treatment. BALB/c mice were treated daily with IMQ cream or control cream on the shaved back skin. Phenotypical presentation of mouse back skin after 6 days of treatment. Erythema, scaling, and thickness of the back skin was scored daily on a scale from 0 to 4. Additionally, the cumulative score (erythema plus scaling plus thickness) is depicted. Symbols indicate mean score ± SD of four mice per group. *epidermal* thickness was measured on the days indicated. Symbols represent mean ± SD for four mice per group

To determine if topical IMQ treatment causes skin inflammation along with psoriasis-like structural characteristics, we applied IMQ cream to BALB/c mice’s shaved back skin for 6 consecutive days. Three days after the start of IMQ application, the back skin of the mice started to display signs of erythema, scaling, and thickening as shown in Fig. 2. The independent scores in a representative experiment are shown in Fig. 3. From days 2–3 onward, inflammation was visible, which continually increased in severity up to the end of the experiment. Mice shaved and treated daily with control cream did not show any sign of inflammation. The naïve group showed no abnormality. As an independent parameter of skin inflammation, we measure thickness in mice. Daily treatment of the thickness of the mice led to significant increases that were measurable from days 5–6 onward, as shown in Fig. 3. The modified PASI score was consistent with the subjective results, as illustrated in Fig. 3. The positive control group showed no enhancement of either the scales, as shown in Fig. 3 or erythema with the maintenance of the scratch behavior The standard topical steroid-treated group showed mild improvement with reduced scale accumulation and erythema darkening. The group treated by a low dose of IL-2 showed moderate improvement as compared to control and naïve groups shown in Fig. 3. Interestingly, the group treated with anti-IL2\IL-2 complex showed decreases after three days of treatment and maximum response after the seventh day from the onset of treatment with the disappearance of the scales and erythema and a decrease in the scratch behavior frequencies (Data not shown), and after 7 days of treatment.

### Anti-IL-2/IL-2 complex showed highly significant decreases in the thickness of the epidermis as compared to the control group

**Fig. 4:**
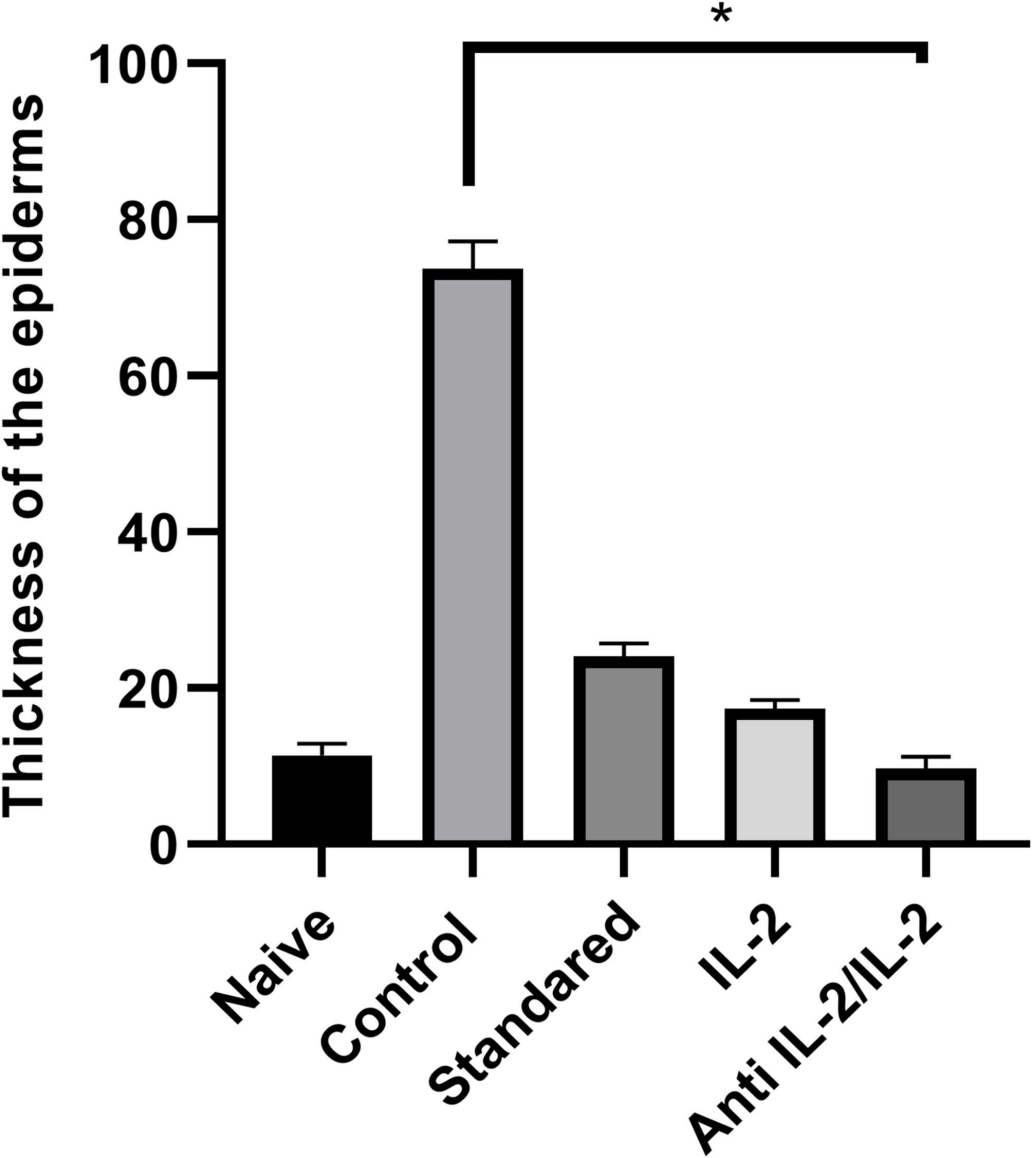
Morphometric analysis of the mean thickness of the epidermis after treatment of mice with PBS (control), bentamethosone (standard group), free IL-2, or anti-IL-2/IL-2, Mice inaïve group are with no psoriasis. Pairwise comparisons statistically significant by the Bonferroni correction (*p*-value < 0.05)

IMQ-treated skin showed increased epidermal thickening in the back skin as shown in Fig. 4. Although the standard and IL-2 groups showed decreases in epidermal thickness, the Group treated with the anti-IL-2/IL-2 complex showed highly significant decreases (*P*-value ≤ 0.005) in epidermal thickness compared to the control group analyzed by ANOVA test, as confirmed by our morphometric study in Fig. 4. After applying the Bonferroni correction, the following pairwise comparisons remain statistically significant (*p* < 0.05). Epidermal thickness showed significant decreases compared to the Anti-IL-2/IL-2 treated group as compared to standard treatment (*p*-value = 0.02). Also, the epidermal thickness showed decreases in the anti-IL-2/IL-2 group as compared to the control (*p*-value = 0.01). On the other hand, the epidermal thickness showed increases in Control as compared to naïve (*p*-value = 0.01). There was no significant difference between anti-IL-2/IL-2 and IL-2 or anti-IL-2/IL-2, and naïve or IL-2 and naive mice (*p*-value = 0.80) in the epidermis thickness.

### Anti-IL-2/IL-2 complex results in increased proliferation and altered differentiation of keratinocytes

**Fig. 5:**
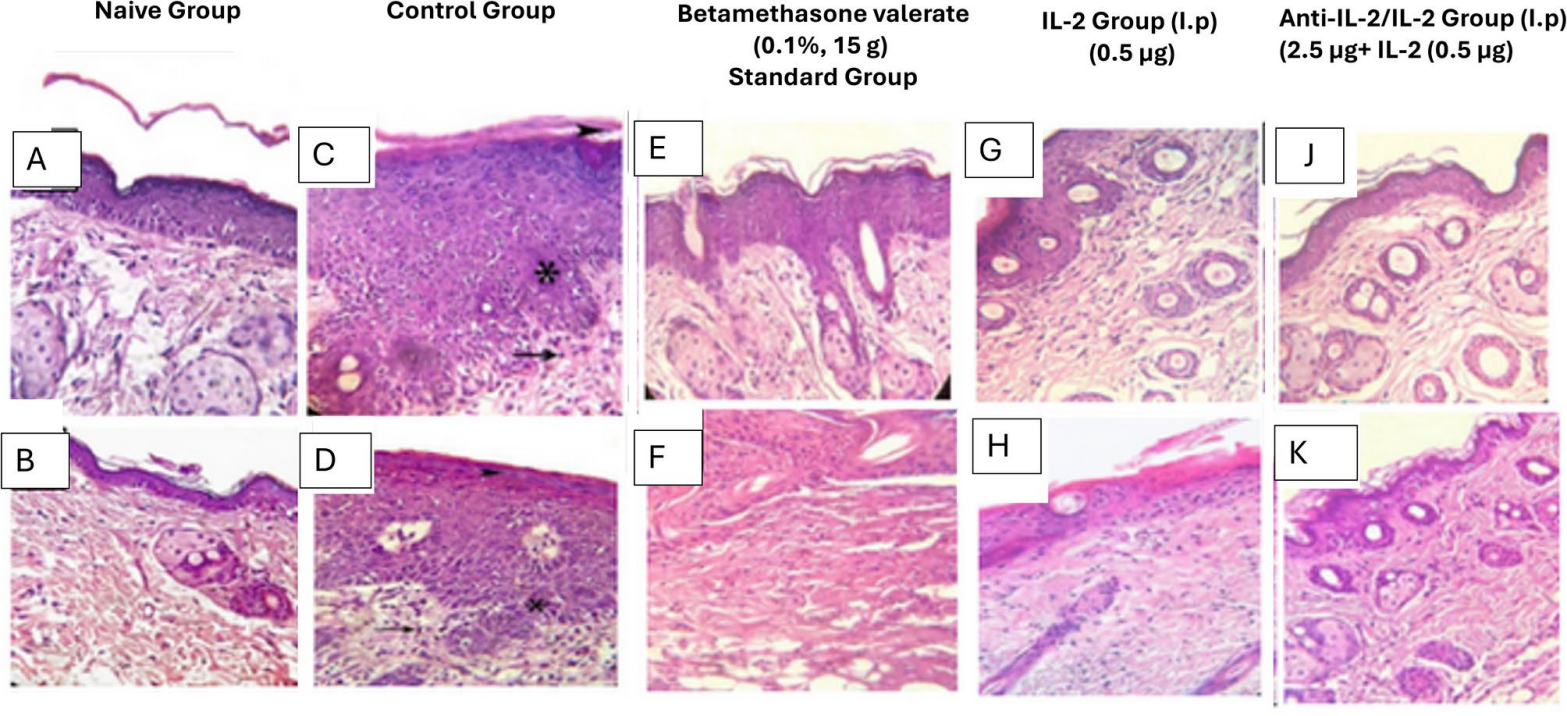
Histopathological examination after treatment of studied group. The naïve group (**A**, **B**) shows normal skin architecture without pathological abnormalities. The positive control group (**C**, **D**) exhibits significant parakeratosis, acanthosis, hyperkeratosis, papillomatosis, and lymphocyte infiltration. Betamethasone treatment (**E**, **F**) leads to mild improvement in these pathological changes.The IL-2 treatment group (**G**, **H**) after seven days shows moderate improvement. The anti-IL-2/IL-2 complex group (**J**, **K**) demonstrates marked improvement with reduced parakeratosis, acanthosis, hyperkeratosis, and lymphocyte infiltration, suggesting a stronger therapeutic effect

Analysis of H&E-stained sections from the naïve group showed no histopathological abnormalities as shown in Fig. 5A and B. While IMQ-treated skin showed acanthosis with exocytosis was prominent in the epidermal layer which was caused by the hyperproliferation of keratinocytes, as increased numbers of keratinocytes in the basal cell layer as shown in Fig. 5C and D. Close examination of H&E-stained sections of back skin showed retention of nuclei in the stratum corneum of IMQ-treated mice indicated with arrows in Fig. 5C and D. Lymphocyte infiltration was profuse all over the dermis. Additionally, there were some degrees of papillomatosis. Munro microabscesses were observed in the keratin layer with a collection of neutrophils.

Groups treated with betamethasone showed mild improvement (*P*-value ≤ 0.005) in parakeratosis, acanthosis, hyperkeratosis, and lymphocyte infiltration as shown in Fig. 5E and F. A low dose of IL-2 group showed the moderate improvement in parakeratosis, acanthosis, hyperkeratosis, and lymphocyte infiltration (*P*-value ≤ 0.005) as shown in Fig. 5G and H. Scaling of the skin is often an indication of parakeratosis, that is, altered epidermal differentiation, a phenomenon typical for psoriasis skin lesions. The groups treated with anti-IL-2/IL-2 complex showed marked improvement in parakeratosis, acanthosis, hyperkeratosis, and lymphocyte infiltration (*P*-value ≤ 0.005) as shown in Fig. 5I and K. in addition, the thickness of epidermis showed improvement as compared to control.

### Representative data illustrating the gating strategy of T_reg_ cells (CD4^+^Foxp3^+^CD25^+^) showed increases in anti IL-2/IL-2 complex group as compared to control

**Fig. 6:**
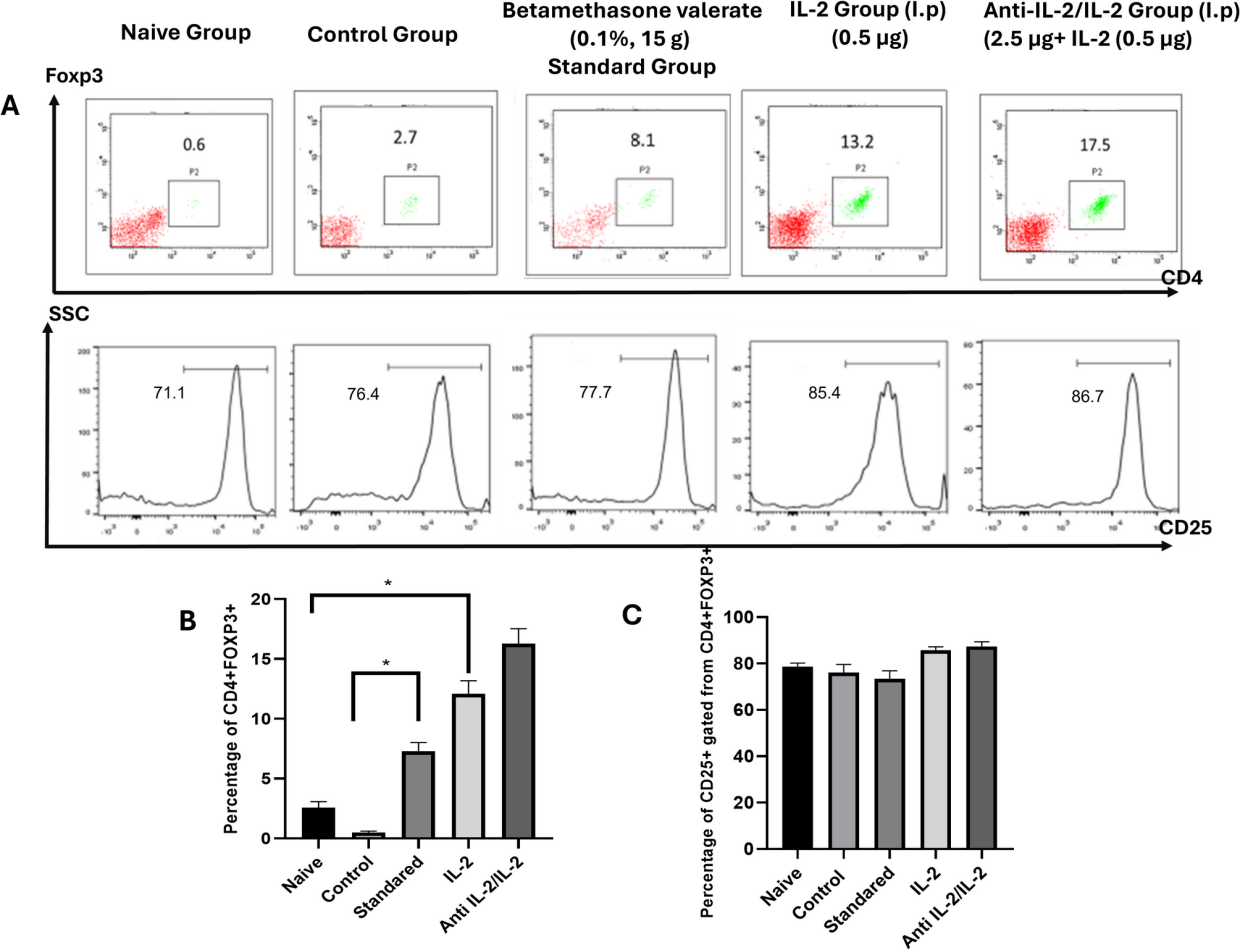
Representative data showing the gating strategy of T_reg_ phenotype CD4^+^ FOXP3^+^ and then CD25^+^ gated from CD4^+^ FOXP3^+^ in the studied groups (**A**). The percentage of CD4^+^ Foxp3^+^CD25^+^ (**B**) CD4^+^FOXP3^+^ (**C**) CD25+ gated from CD4^+^FOXP3^+^ in the studied group

We found that the percentage of CD4^+^Foxp3^+^ showed highly significant increases in the anti-IL-2/IL-2 complex group (7.8 ± 4.40), as well as, the group treated with a low dose of IL-2 (13.83 ± 2.61) as compared to naive and control groups (1.47 ± 0.81) (*P* value ≤ 0.001). However, the standard therapy group showed decreases in the percentage of CD4^+^ FOXP3^+^ (4.47 3.17) as compared to the control group (0.6 ± 3.54), as shown in Fig. 6. Moreover, the percentage of CD25 ^+^ gated on CD4^+^ FOXP3^+^ in all studied groups showed a highly significant increase in anti-IL-2/IL-2 complex and low dose of IL-2 treated group with percentage of (88.03 ± 1.533 and 82.27 ± 2.72), respectively (*P* value ≤ 0.001) as compared to the control group with (78.97 ± 1.45). However, there was no significant change in the standard treated group (75.40 ± 2.46), as compared to naïve and control groups, as shown in Fig. 6A-C.

## Discussion

Psoriasis vulgaris is one of many autoimmune diseases that cause changes to the skin’s constituents, typically resulting in undesirable topical symptoms [31]; however, the mechanism of its pathogenesis remains fully understood. The imbalance of T_reg_ cells and effector T cells represents one potential mechanism: a low dose of IL-2 is important. Given that the anti-IL-2/IL-12 complex is considered an immune modulator for antigen-activated lymphocyte proliferation, this study aimed to compare the immunophenotypic, clinical, and histological effect of anti-IL2/IL-2 complex with a low dose of IL-2 on psoriasis-like skin inflammation induced by imiquimod. After 7 days from the onset of treatment, we found that treatment of mice with anti-IL-2/IL-2 complex decreased the thickness of the epidermis as compared to other groups. IMQ-induced hyperkeratosis, parakeratosis and mild papillomatosis with the retained nuclei in the keratin layer, whereas acanthosis with exocytosis was prominent in the epidermal layer. Lymphocyte infiltration was profusely all over the dermis. Additionally, there were some degrees of Munro micro abscesses were observed in the keratin layer with a collection of neutrophils. The group treated with standard betamethasone cream showed mild improvement clinically, histopathological and no significant difference between this group and the naïve and positive control groups. In line with previous studies, Epidermal changes based on keratinocyte showed hyperproliferation and altered differentiation. Papillomatosis (regular and symmetrical extension of rete ridges, separated by elongated dermal papillae) and presence of inflammatory cells including T cells, DC, and neutrophils, and) a functional role for T cells as well as, altered vascularity [31, 32].

Currently, there are 3 approved IL-23 inhibitors for psoriasis treatment: risankizumab-rzaa (Skyrizi; Boehringer Ingelheim and AbbVie), guselkumab (Tremfya; Janssen Biotech, Inc), and tildrakizumab-asmn (Ilumya; Sun Pharma). The 3 approved IL-17 inhibitors are secukinumab (Cosentyx; Novartis), ixekizumab (Taltz; Eli Lilly and Company), and brodalumab (Siliq; Ortho Dermatologics). There is also an oral phosphodiesterase-4 inhibitor called apremilast (Otezla; Amgen) approved by the FDA [26]. In recent studies, secukinumab showed rapid early-onset efficacy, Strober said; however, IL-23 inhibitors demonstrated greater long-term efficacy after 1 year. Secukinumab is as fast as or maybe even a little faster than the IL-23 inhibitors as a group. But over the long haul, IL-23 numbers show better sustained efficacy. Additionally, tumor necrosis factor (TNF) inhibitors are, in his opinion, as efficacious as IL-17 inhibitors for psoriatic arthritis [33].

For a long time, it was thought that IL-2 is an inflammatory cytokine and its presence is responsible for the autoimmune reaction [34]. Then, the IL-2 paradox emerged after discovering that the IL-2 gene knockout in mice caused the flourishing of the reaction. Therefore, efforts have been exerted, showing that ultra-dose of IL-2, either alone or complexed with anti-IL2 Abs, can have a potential effect in selective, in vitro, activation and enriching of T_reg_ cells in the clinical studies [27]. Our results showed that anti-IL-2/IL-2 complex yielded the highest response either clinically with the disappearance of the scales and erythema and decrease in the scratch behaviour frequencies, or microscopically with the enhancement of lymphocytes infiltration, hyperkeratosis, acanthosis, the disappearance of parakeratosis and Munro microabscesses. Furthermore, the percentage of CD4^+^ Foxp3^+^ showed highly significant increases in the anti-IL-2/IL-2 complex treated group (7.8 ± 4.40), as well as, the group treated with a low dose of IL-2 (13.83 ± 2.61) as compared to naive and control groups (1.47 ± 0.81) (*P* value ≤ 0.001). However, the standard therapy group showed decreases in the percentage of CD4^+^ FOXP3^+^ (4.47±3.17) as compared to the control group (0.6 ± 3.54). It is consistent with previous studies that showing flow cytometry using the draining lymph nodes harvested on Day 8 revealed an increase in CD4^+^Foxp3^+^ cells in draining lymph nodes (LN) post imiquimod application. In addition, the immunohistochemical analysis for T_reg_ cells using back skin (epidermis and dermis) harvested on Day 8 showed an increased number of T_reg_ cells in imiquimod-applied mouse (*P* = 0.006) [35]. A recent study showed that IL-2 could trigger both CD8^+^ T cells and T_reg_, however, IL-2 induced signaling occurs at markedly lower concentrations of IL-2 in T_Reg_ cells than in effector T cells or NK cells. T_reg_ cells have a 10–20 folds lower activation threshold for IL-2 than effector T cells [36]. The higher efficacy of anti-IL-2/IL-2 complex than low dose of IL-2 alone could be attributed to that the anti-IL-2 binds to a specific site on IL-2 that is appropriate for its interaction with CD8^+^ memory T cells, but maintains its binding to the high affinity α-chain (CD25) which is expressed at the highest levels on T_reg_ [37, 38]. Thus, the effect of IL-2 when bind to the complexes is preferentially directed towards CD25 high regulatory cells rather than CD8 ^+ ^T memory cells. Anti-IL-2/IL-2 complex has been proven to improve several immune disorders in mice owing to the extensive expansion and activation of T_reg_ in vivo [39]. Moreover, the complex of anti-IL-2/IL-2 extends the half-life than when IL-2 is used alone [40].

To our knowledge, this is the first study to assess the effect of either low dose of IL-2 alone or in combination with anti-IL-2 in the management of psoriasis-like skin inflammation induced in Balb/c mice by imiquimod. In 2010, Liu et al. showed that injection of immune complexes composed of the cytokine IL-2 and anti-IL-2 mAb induced an effective and sustained expansion of T_reg_ via peripheral proliferation of CD4^+^Foxp3^+^CD25^+^ cells and peripheral conversion of CD4^+^Foxp3^−^CD25^− ^cells. The expanded T_reg_ potently suppressed autoreactive T- and B-cell responses to acetylcholine receptors [39, 41] and attenuated the muscular weakness in an experimental mice model Another study initiated in mice, using IL-2 complexes, showed that a short course of daily injections increased T_reg_ numbers by 7–15-fold [40], while CD8^+^ T and NK cells were not considerably affected [42]. Rosenzwajg et al., in 2018, assessed the use of low dose of IL-2 in 11 types of rheumatological diseases and reported the universal safety, biological efficacy and possible clinical efficacy of low dose of IL-2 across a group of very heterogeneous diseases including, psoriasis, rheumatoid arthritis (RA), ankylosing spondylitis (AS), SLE, Behçet’s disease, granulomatosis with polyangiitis, Takayasu’s disease, Crohn’s disease (CD), ulcerative colitis (UC), autoimmune hepatitis and sclerosing cholangitis [43, 44]. The safety profile of low dose of IL-2 across the different diseases and across various background treatments was very good. There have been no serious adverse event related to treatment. To be noted that, in our results, low dose of IL-2 also significantly improved the Psoriasis-like skin inflammation induced by imiquimod. Jeffery et al., in 2017, investigated the effects of very low dose IL-2 the subsequent survival and function of T_Reg_ and T effector cells from the blood and livers of patients with autoimmune liver diseases associated with increased expression of FoxP3^+^ and CD25^+^ and the anti-apoptotic protein Bcl-2 in T_reg_ [45].

In conclusion, anti-IL-2/ IL-2 complex yielded the highest response either clinically, or microscopically. Furthermore, the percentage of CD4^+^ Foxp3^+^ showed highly significant increases in the anti- IL-2/IL-2 complex treated group, as well as, the group treated with a low dose of IL-2 as compared to naive and control groups. Anti IL-2/IL-2 complex therapy effectively ameliorated the clinical manifestations of psoriasis, with no apparent side effects, providing a new strategy for treating psoriasis. Further studies, either clinical or preclinical, should begin assessing the role of anti-IL-2/IL-2 complex in comparison with low doses of IL-2 in the management of Ps patients and in comparison to other biologics modalities. Also, our study could be rehearsed with the use of a higher dose of both therapies and should be assessed in other psoriasis mouse models.

## Data Availability

All data generated or analyzed during this study are included in this published article. More detailed data is available from the corresponding author upon reasonable request.
